# *Angiostrongylus cantonensis* cathepsin B-like protease (Ac-cathB-1) is involved in host gut penetration

**DOI:** 10.1051/parasite/2015037

**Published:** 2015-12-18

**Authors:** Ying Long, Binbin Cao, Liang Yu, Meks Tukayo, Chonglv Feng, Yinan Wang, Damin Luo

**Affiliations:** 1 School of Life Sciences, Xiamen University Fujian 361102 P.R. China; 2 State Key Laboratory of Cellular Stress Biology, Xiamen University Fujian 361102 P.R. China; 3 Medical College, Xiamen University Fujian 361102 P.R. China

**Keywords:** Lentiviral expression, *Angiostrongylus cantonensis*, Recombinant Ac-cathB-1, Protease

## Abstract

Although the global spread of the emerging zoonosis, human angiostrongyliasis, has attracted increasing attention, understanding of specific gene function has been impeded by the inaccessibility of genetic manipulation of the pathogen nematode causing this disease, *Angiostrongylus cantonensis*. Many parasitic proteases play key roles in host-parasite interactions, but those of *A. cantonensis* are always expressed as the inactive form in prokaryotic expression systems, thereby impeding functional studies. Hence, a lentiviral system that drives secreted expression of target genes fused to a Myc-His tag was used to obtain recombinant Ac-cathB-1 with biological activity. Although this class of proteases was always reported to function in nutrition and immune evasion in parasitic nematodes, recombinant Ac-cathB-1 was capable of hydrolysis of fibronectin and laminin as well as the extracellular matrix of IEC-6 monolayer, so that the intercellular space of the IEC-6 monolayer increased 5.15 times as compared to the control, while the shape of the adherent cells partly rounded up. This suggests a probable role for this protease in intestinal epithelial penetration. The inhibition of Ac-cathB-1 enzymatic activity with antiserum partly suppressed larval penetration ability in the isolated intestine. Thus, an effective system for heterologous expression of parasite proteases is presented for studying gene function in *A. cantonensis*; and Ac-cathB-1 was related to larval penetration ability in the host small intestine.

## Abstract (fr)

Bien que la propagation mondiale de l’angiostrongylose humaine, une zoonose émergente, attire une attention croissante, la compréhension de la fonction de gènes spécifiques a été entravée par l’inaccessibilité de la manipulation génétique du nématode pathogène de cette maladie, *Angiostrongylus cantonensis*. Beaucoup de protéases parasitaires jouent des rôles clés dans l’interaction hôte-parasite, mais celles *d’A. cantonensis* sont toujours exprimées sous forme inactive dans les systèmes procaryotes d’expression, empêchant ainsi des études fonctionnelles. Par conséquent, un système lentiviral qui permet l’expression sécrétée de gènes cibles fusionnés à un marqueur Myc-His a été utilisé pour obtenir de l’Ac-cathB-1 recombinante ayant une activité biologique. Bien que cette classe de protéases ait toujours été rapportée comme impliquée dans l’alimentation et l’évasion immunitaire chez les nématodes parasites, l’Ac-cathB-1 recombinante était capable d’hydrolyser la fibronectine et la laminine, ainsi que la matrice extracellulaire de monocouches IEC-6, de sorte que les espaces intercellulaires des monocouches IEC-6 étaient augmentés de 5.15 fois par rapport au contrôle, tandis que la forme des cellules adhérentes devenait partiellement arrondie. Ceci suggère un rôle de cette protéase dans la pénétration de l’épithélium intestinal. L’inhibition de l’activité enzymatique d’Ac-cathB-1 par un antisérum a supprimé en partie la capacité de pénétration des larves dans l’intestin isolé. Ainsi, un système efficace pour l’expression hétérologue de protéases de parasites est présenté pour l’étude de la fonction des gènes chez *A. cantonensis*, et Ac-cathB-1 a été liée à la capacité de pénétration de l’intestin grêle de l’hôte par des larves.

## Introduction

Human angiostrongyliasis is an emerging zoonosis caused by *Angiostrongylus cantonensis* (Chen, 1935). Recently, this disease has spread worldwide from its traditional endemic regions due to increasing global trade and travel, and is now one of the major threats to public health [6, 34]. The rat is the definitive host of *A. cantonensis*. The first-stage larvae (L1) of this worm are passed via infected rat feces and develop into the third-stage larvae (L3) in the intermediate hosts, such as snails and slugs [15]. Humans and rats are infected by consuming raw or undercooked intermediate hosts, infected transport hosts, or contaminated vegetables [1, 11]. When the infective larvae (L3) are swallowed, they reach the host small intestine and penetrate through the intestinal wall into the systemic circulation. The larvae then migrate to the central nervous system where they mature, and finally settle in the pulmonary artery in rats. However, in non-permissive hosts, they cause diseases and are unable to finish their life cycle. Small intestine penetration is found to be important for L3 to infect hosts. However, the detailed molecular events during this process are not yet very clear.

Numerous cathepsin Bs of parasitic nematodes have been proposed to be involved in diverse biological processes, such as nutrition and immune evasion [31]. In our previous work, recombinant Ac-cathB-1 (rAc-cathB-1) was expressed in an insoluble form without correct folding and modification in *E. coli*, and this cathepsin B-like cysteine protease of *A. cantonensis* was predicted to have functions in parasite-host interactions in addition to its function in digestion [24, 38]. Recently, RNAi and certain gene manipulations have been achieved in diverse parasitic organisms [18, 26, 29], but many other species were proven refractory to effective genetic manipulation [21, 33], which hindered the functional understanding of the specific genes. The manufacture of pure gene products in a heterologous system makes it possible to analyze their properties and function *in vitro*. Although some genes were expressed in a bioactive form for planned analysis in prokaryotic expression systems [5, 7], other genes were frequently expressed as inclusion bodies that need solubilization and refolding [27, 37]. Mammalian cell-based expression systems produce proteins with biological activity due to the capacity of handling complex post-translational modifications and folding into the native conformation. The lentiviral vector (LV) derived from human HIV-1 has been widely applied in this field due to its ability to transduce and integrate exogenous genes into the genome of infected cells [2], which provided an efficient alternative to plasmid transfection. Thus, mammalian cell lines can be created for stable expression; however, collection of the target protein from cell lysates is unfavorable for downstream purification.

Therefore, the aims of the present work were to (1) develop a modified LV-based expression system for harvesting the target protein, Ac-cathB-1, from the mammalian cell culture supernatant, (2) evaluate the hydrolytic activity of rAc-cathB-1, thus presenting an effective system for heterologous expression of parasite genes, and (3) explore the function of Ac-cathB-1 in larval gut penetration.

## Materials and methods

### Nematode

*A. cantonensis* was maintained in Sprague Dawley (SD) rats. The sugar flotation technique was used to collect L1 from infected rat feces after 45 d. Fresh *A. cantonensis-*positive rat feces were added to water, mixed into a paste, and lettuce was coated with the paste and fed to apple snails (*Pomacea canaliculata*) to cause infection. After 3 weeks of being infected, the snails were cut into small pieces and digested overnight in digestive fluid (0.7% pepsin in 0.5% HCl). *A. cantonensis* L3 were collected under a dissecting microscope for later experiments.

The SD rats were managed and housed in the Xiamen University Laboratory Animal Center. The use of mammals in this study was reviewed and permitted by the Committee for the Care and Ethics of Laboratory Animals of Xiamen University according to the Regulations for the Administration of Affairs Concerning Experimental Animals (approved by the State Council of the People’s Republic of China), with the laboratory animal usage License Number XMULAC2012-0122.

### Construction of lentiviral vector

An IgK signal peptide (I, from pSecTag2B vector) and a Myc-His encoding sequence (MH, from pcDNA3.1/*myc*-His A vector) were amplified with primer pairs P1 and P2, respectively (P1F: GCTAG-CCACCATGGAGACAGACACAC, P1R: CTCGAGAAGCTTTTCGAAACCGGTTCTAGATCGTA-CGGCGCGCCTGGC, *Nhe*I, *Xho*I-*Hind*III, and *Xba*I restriction sites are underlined; P2F: AAGCTTGGATCCGAACAAAAACTCATCTCAGAAGAGG, P2R: CTCGAGAGATCTTCAATG-GTGATGGTGATGA; *Hind*III-*BamH*I and *Xho*I-*Bgl*II restriction sites are underlined), and ligated into pEASY-T5 Zero cloning vector (TransGen Biotech) successively to form a pEASY-T5-IMH plasmid by *Hind*III and *Xho*I restriction sites. Subsequently, the green fluorescent protein (GFP) encoding sequence in the pBobi-GFP vector, which was coordinately expressed with puromycin resistance by the regulation of the IRES sequence [17], was replaced by an IMH fragment using an isocaudamer technique, resulting in the creation of a modified LV pBobi-IMHIP (IgK SP-Myc-His-IRES-Puro). To generate the pBobi-cathB1 vector, the region excluding the native signal peptide (SP) encoding sequence in the *Ac-cathB-1* open reading frame (Ser-27 to Phe-394) was amplified by high-fidelity PCR with primers P3 (P3F: TCTAGAAGCGATTCGTCAGAAGACAACGAC, P3R: GGATCCGAAGTCGTCGTCTTCCCATG-CAT; *Xba*I and *BamH*I restriction sites are underlined), and incorporated into the expression cassettes of pBobi-IMHIP vector by *Xba*I and *BamH*I restriction sites.

### Creation and identification of stably expressed cell lines

As pBobi-cathB1 did not carry a reporter gene, pBobi-GFP served as a positive control to show transfection and screening efficiency. The recombinant virus was packaged effectively as previously described [14] by polyethyleneimine (PEI, Sigma-Aldrich) mediated cotransfection of 293T cells (CRL-11268, ATCC) with lentiviral transfer vectors (pCMV-VSV-G and pHR) and main plasmid (pBobi-GFP or pBobi-cathB1). Briefly, the 293T cells were seeded and cultured to 80% confluence on the second day with DMEM supplemented with 10% fetal bovine serum (FBS, HyClone). A mixture of plasmids, 12 μg of main plasmid, 4 μg of pHR, and 4 μg of pCMV-VSV-G, was transfected into every 10-cm dish of cells with PEI. The medium was replaced with fresh DMEM containing 2% FBS 8 h later, and the lentivirus-containing culture supernatant was collected 2 d later. To transfer the target gene, the recombinant virus was used to infect a fresh monolayer of 293T cells in the presence of 8 μg/mL polybrene (CHEMICO). After 12–24 h, the medium was washed off, and cell line selection was performed using puromycin at a final concentration of 2 μg/mL from the second day for no less than 2 weeks. The puromycin-resistance screening continued until cells without green fluorescence were undetected in the 293T-GFP.

The establishment of cell lines was confirmed by immunofluorescent (IF) staining, which was performed as described previously with slight modification [32]. Briefly, two samples were rinsed and fixed with precooled ethanol (10 min at −40 °C). A solution of 0.25% (v/v) Triton X-100 in phosphate-buffered saline (PBS, pH 7.4; 137 mM NaCl, 2.7 mM KCl, 10 mM Na_2_HPO_4_, 1.8 mM KH_2_PO_4_) was used for permeabilization for 15 min. After blocking with normal goat serum (TransGen Biotech, 10%, v/v in PBS), incubations with primary antibody (anti-Myc, TransGen Biotech, 1:1000) and an AF488-coupled anti-mouse secondary antibody (1:1000) were performed successively. Subsequently, DAPI (Sigma-Aldrich) was used for nuclear staining. Stained cells were analyzed with a fluorescence microscope and the images were captured by a digital camera and Image-Pro Plus 6.0 (IPP 6.0, Media Cybernetics) software.

Success of ectopic expression of Ac-cathB-1 was confirmed at the mRNA and protein levels. Total RNA of two cell lines was extracted using TRIzol reagent (Life Technologies) following the manual. All RNA samples (1 μg) were reverse transcribed into the first-strand cDNAs using PrimeScript II 1st Strand cDNA Synthesis Kit (TaKaRa) for semi-quantitative reverse transcription (RT)-PCR according to the manufacturer’s instructions. The RT-PCR was carried out according to the previous report [9], with the primers P4 (P4F: ATCATGTTGGGCATTCGG, P4R: GCATTTCGGTG-TTGGGTA); and human *Actb* served as the reference (P5F: CCCAGAGCAGTCTTTCCTTCCA, P5R: CCATAGGGTATTTCAGCGTTAG). The amplified PCR products were analyzed on a 1.5% agarose gel with ethidium bromide staining and captured digitally using the Molecular Imager Gel Doc XR+ System (Bio-Rad). Equal amounts of protein from these two cell lines were analyzed by 12% SDS-PAGE and transferred onto a PVDF membrane (Millipore). The membrane was subsequently washed twice with TBST (Tris-buffered saline, TBS, 150 mM NaCl, 10 mM Tris, pH 8.0; TBST, TBS containing 0.05% (v/v) Tween-20) and blocked with 5% (w/v) skimmed milk for 1 h. After incubating with the homemade polyclonal antiserum against prokaryotic rAc-cathB-1 (1:1000) and horseradish peroxidase (HRP)-conjugated anti-mouse secondary antibody (1:5000, Sigma-Aldrich) successively, electrogenerated chemiluminescence (ECL, Thermo) was applied to visualize the target protein and the bands were captured digitally using the Molecular Imager Gel Doc XR+ System (Bio-Rad). The homemade antiserum was prepared by immunized mice and was available for immunohistochemical staining and western blot assay in our previous work [38].

### Expression and purification of rAc-cathB-1

The 293T-cathB1 cells were cultured to 90% confluence and the medium was replaced with DMEM containing 2% FBS, and the cell culture supernatant was collected every 24 h; repeated three times. His-tagged Ac-cathB-1 in the cell culture medium was purified using immobilized metal affinity chromatography (Ni-NTA resin, GE Healthcare) followed by gel filtration. One liter of culture supernatant was harvested and concentrated using Amicon Ultra centrifugation tubes (Millipore) with a molecular weight cut-off of 10 kDa and diluted with binding buffer (20 mM Tris, 200 mM NaCl, 20 mM imidazole, pH 8.0). The sample was then loaded onto the Ni-NTA resin equilibrated with the binding buffer and eluted with elution buffer (20 mM Tris, 200 mM NaCl, 250 mM imidazole, pH 8.0). Samples of column flowthrough, binding buffer wash, and eluate were collected. After a buffer exchange, the eluate was loaded onto a Superdex 75 gel filtration column (Amersham Biosciences) equilibrated with 50 mM sodium phosphate buffer (PB, pH 8.0) and fractionated using an Akta chromatography system (GE Healthcare) at 4 °C. Subsequently, all the collected samples were analyzed by 12% SDS-PAGE with Coomassie brilliant blue staining, and the fractions containing target protein pooled and concentrated.

### Identification of rAc-cathB-1

The sample of the purified protein was analyzed by western blot using a commercial antibody against Myc (TransGen Biotech, 1:1000) and the homemade polyclonal antiserum (1:1000) against prokaryotic rAc-cathB-1 as the primary antibody respectively. Afterward, tandem mass spectrometry (MS) assay of rAc-cathB-1 was performed in IDA (information dependent acquisition) mode according to the manual. After trypsinization of the sample, peptide mixture was purified, followed by desalination and lyophilization, and dissolved in 2% acetonitrile (ACN) containing 0.1% formic acid (FA). MS analysis was performed on a TripleTOF 5600 mass spectrometer (AB SCIEX) coupled to a NanoLC Ultra 2D Plus (Eksigent) HPLC system. The peptides first bound to a 5 mm × 500 μm trap column packed with Zorbax C18 5-μm 200-Å resin using 0.1% FA/2% ACN in H_2_O at 10 μL/min for 5 min, and then separated using a 60-min gradient from 2 to 35% buffer B (buffer A, 0.1% FA, 5% Dimethyl sulfoxide [DMSO] in H_2_O, buffer B, 0.1% FA, 5% DMSO in Acetonitrile [ACN]) on a 15 cm × 75 μm in-house pulled emitter-integrated column packed with Magic C18 AQ 3-μm 200-Å resin. The raw data files (*.wiff) were searched with ProteinPilot Software v. 4.1.46 beta (AB SCIEX) using the Paragon and Progroup Algorithms against the Uniprot *Mus musculus* database (canonical and isoform sequence data, containing 50,402 sequences, downloaded in April 2011 from http://www.uniprot.org/) with common contaminants included.

### Activation of rAc-cathB-1

Purified rAc-cathB-1 was activated according to the protocol described previously [12]. One volume of activation solution (10 μg/mL pepsin in 0.5 M sodium phosphate, pH 3.0; pepsin from porcine gastric mucosa, Sigma-Aldrich) was added to two volumes of rAc-cathB-1 solution (0.2 mg/mL in phosphate-buffered saline, pH 7.0). Two volumes of rAc-cathB-1 added to one volume of reference solution (0.5 M sodium phosphate, pH 3.0) served as the control. The mixtures were incubated at 37 °C for 30 min, and the reactions were stopped by addition of pepstatin A (Sigma-Aldrich) to a final concentration of 1 mM.

The chemical Z-Arg-Arg-7-amido-4-methylcoumarin hydrochloride (Z-RR-AMC, Sigma-Aldrich) is a fluorescent substrate for cathepsin B. Enzyme activity of the two treatment groups described above was investigated by measuring the fluorescence of the released AMC with excitation and emission wavelengths of 355 and 460 nm, respectively [4]. The stock buffer used in this assay was 100 mM sodium citrate (pH 5.5) containing 5 mM DTT, and the reaction was initiated by adding substrate to a final concentration of 50 μM. After incubation for up to 30 min at 37 °C, the fluorescence of released AMC was measured and data were presented as relative activities, where activity of the control was taken as 1.

### The pH-dependence profile of activated rAc-cathB-1

The pH-dependence profile of activated rAc-cathB-1 was measured using Z-RR-AMC. The assay was performed using constant ionic strength acetate-4-morpholineethane sulfonic acid (MES)-Tris buffers (100 mM acetate, 200 mM Tris, and 100 mM MES, 4 mM Na_2_EDTA, pH 4.0–9.0) containing 5 mM DTT. In this assay, 0.5 μg of rAc-cathB-1 was activated and added to buffers with successive pH values, and the reaction was started by the addition of the substrate at a final concentration of 50 μM. After incubation for 30 min at 37 °C, the fluorescence was measured as described above and the data were presented as relative activities of activated rAc-cathB-1, where the highest activity at the pH optimum was taken as 100%.

### Hydrolytic activity analysis of activated rAc-cathB-1

The hydrolysis of various substrates was determined within the incubation buffer, 100 mM sodium citrate containing 5 mM DTT (pH 6.0), with or without the cathepsin B inhibitor E64 (ApexBio) at a final concentration of 10 μM. One microgram of purified rAc-cathB-1 was activated and incubated with 10 μg of the respective substrates including type I collagen, laminin, and fibronectin (Sigma-Aldrich) for 12 h. Ten micrograms of the respective substrates, only adding equal volumes of PBS, served as the controls. After incubation, samples were analyzed on a 5% SDS-PAGE gel followed by Coomassie brilliant blue staining.

A rat intestine epithelial cell line, IEC-6, is useful to analyze the interaction between parasites and the host intestine [19, 35]. It was cultured on a glass coverslip to 90% confluence before the replacement of fresh DMEM (pH 7.0, to mimic the neutral pH environment of the lumen of the small intestine) medium without serum. Activated rAc-cathB-1 with or without 10 μM E64 was added to the medium to a final concentration of 20 μg/mL, and medium containing PBS in the place of rAc-cathB-1 served as the control. After incubating for 2 h, the coverslip was taken out for IF staining assay against laminin (Sigma-Aldrich, 1:500). Briefly, coverslips were rinsed with precooled phosphate-buffered saline (PBS, pH 7.4) and fixed as above. Incubation with Triton X-100 (0.25%, v/v in PBS) for 15 min was performed to permeabilize the cells. Subsequently, incubation with the primary and AF488-coupled anti-rabbit secondary antibodies (1:1000) was performed after blocking with normal goat serum (10%, v/v in PBS), and nucleus of cells was stained with DAPI. All fluorescent images of the control and experiment were captured with the same exposure setting and illumination using an Olympus IX71 inverted fluorescence microscope. To quantify the variation in the cell sheets, three visual fields were randomly selected in each of the separate triplicate samples. After merging of images, we selected the light object using threshold and segmentation tools of IPP 6.0 software, and then the dark areas representing the intercellular gap could be counted using the automatic measurement tool.

### Effect of antiserum on hydrolytic activity

Certain antibodies are capable of blocking the ligand-receptor binding reactions of their target proteins [13]. In hookworm, this method was used for studying the role of *Ancylostoma caninum* MTP-1 protease in skin invasion [36]. Total protein concentrations of the homemade mouse antiserum against prokaryotic rAc-cathB-1 (positive serum, referred to above) and normal mouse serum (negative serum) were measured using BCA Protein Assay Kit (Pierce). One microgram of rAc-cathB-1 was activated and incubated with the positive serum (6.0 μg) or negative serum for 30 min, respectively, prior to assessment of the degradation of Z-RR-AMC. Activated rAc-cathB-1 without serum treatment served as the control. Hydrolysis of the substrate was performed and calculated as above to investigate the effect of positive serum on hydrolytic activity of activated rAc-cathB-1.

### Effect of antiserum on larval gut penetration

The role of Ac-cathB-1 in larval gut penetration was investigated by inhibiting the enzymatic activity of this protease with the positive serum according to previous works [20, 36]. Two hundred L3 larvae were pretreated with the undiluted positive serum, the undiluted negative serum, or PBS for 30 min, respectively. Fresh small intestines were excised from rats, washed with sterilized Tyrode’s solution, and sectioned into 2 cm segments. Either end of the intestinal segments was clamped and ligated to form rat gut sacks. The three groups of larvae were then separately injected into lumens of rat gut sacks and kept in sterilized Tyrode’s solution for 3 h to determine the alteration of larval gut penetration. Each trial was conducted in triplicate and the numbers of larvae remaining in the gut lumen were counted.

### Statistics

All the assays were performed in triplicate at least and the values were expressed as the mean ± standard deviation (*SD*). Significant differences between groups were analyzed by *t*-test or one-way analysis of variance (ANOVA) followed by Duncan’s multiple comparison test with SPSS 13.0 (SPSS, Inc.), with a *P* value < 0.05 considered statistically significant.

## Results

**Figure 1.**
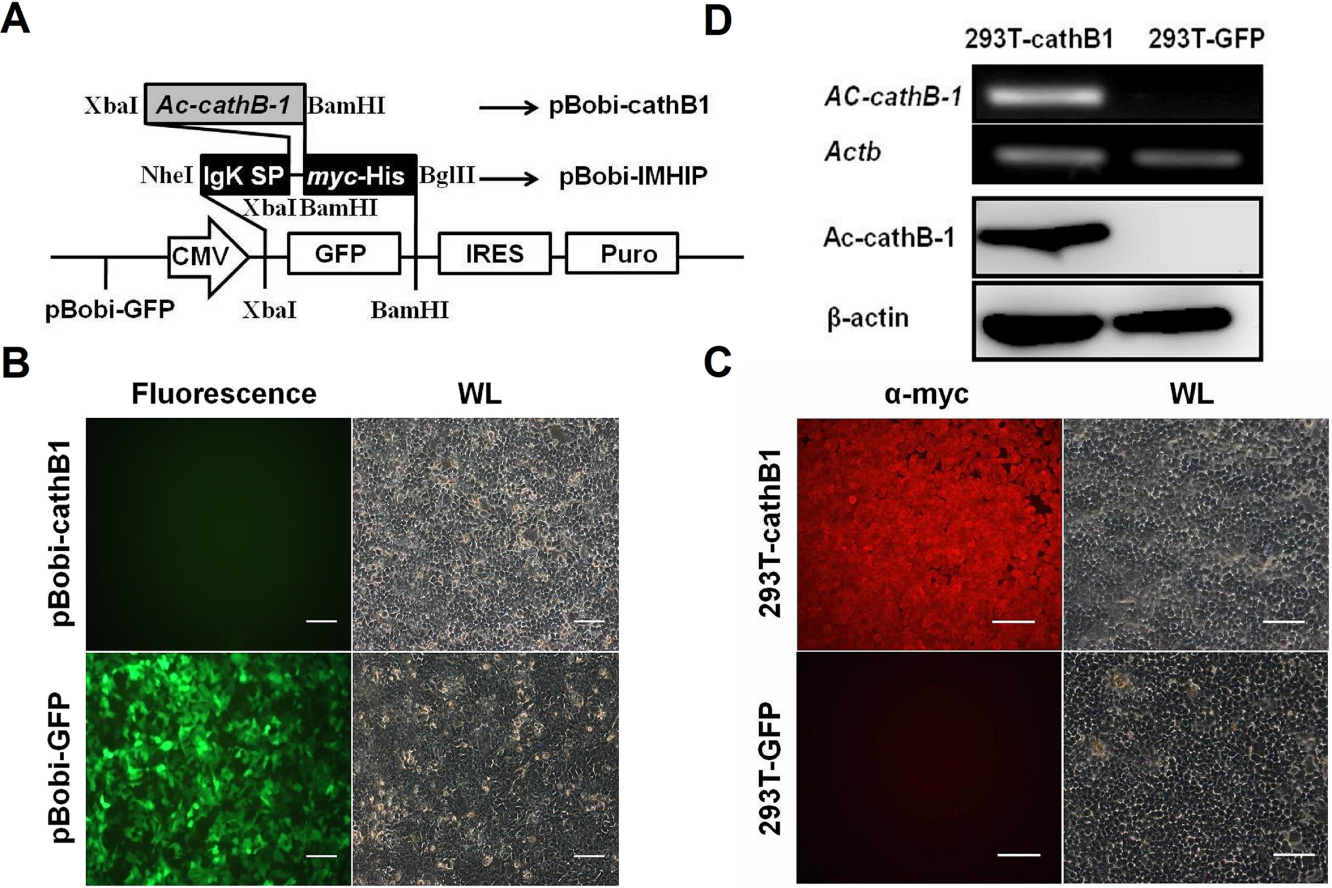
Selection and identification of the stably expressed cell line. (A) Schematic diagram of vector construction. Replacement of the GFP with a sequence containing 5′ IgK SP and 3′ *myc*-His sequence using an isocaudamer technique resulted in the creation of pBobi-IMHIP vector. Lentiviral vector pBobi-cathB1 was generated by insertion of *Ac-cathB-1* coding sequence excluding SP into the pBobi-IMHIP vector by XbaI and BamHI restriction sites. (B) Lentiviral packaging of pBobi-cathB1 and pBobi-GFP. Under the fluorescence microscope, more than 90% of cells in the pBobi-GFP transfected group displayed green fluorescence. (C) Assessment of the establishment of 293T-cathB1. Anti-Myc IF staining was performed in the two cell lines. All cells in 293T-cathB1 showed red fluorescence representing 100% positive, while all cells in 293T-GFP showed no fluorescence under the red fluorescent filter due to the lack of myc-tag expression. (D) Tests for rAc-cathB-1 expression. Total RNA and protein samples were extracted from two groups of cells. Results of RT-PCR (top two panels) and western blot (lower two panels) showed that rAc-cathB-1 was highly expressed in 293T-cathB1 at both mRNA and protein levels. *Actb*, RT-PCR control and *β*-actin, loading control for western blot; WL, white light; and bar = 100 μm.

In order to stably express rAc-cathB-1 with bioactivity, we rebuilt the LV so that the insertion of *Ac-cathB-1* lacking the sequence of native SP was flanked by 5′ IgK SP and 3′ myc-His tags (Fig. 1A). Thus, the desired expression product fused to an N-terminal myc-His tag was expected to be secreted into the culture medium with a theoretical molecular weight of 45.6 kDa, which facilitates planned downstream purification.

We utilized the PEI-mediated method to cotransfect 293T cells with three proportional plasmids for lentivirus package. Under the fluorescence microscope, cells transfected with pBobi-GFP displayed an intense green fluorescence with a transfection efficiency higher than 90% (Fig. 1B), indicating an effective transfection. The recombinant viruses were then harvested and used to infect fresh 293T cells. Stable cell lines were generated after infection and puromycin-resistance screening. Immunofluorescent staining against the myc-tag was performed to analyze the rate of positive cells, and all cells of 293T-cathB1 exhibited red fluorescence, while 293T-GFP cells were negative (Fig. 1C), implying the successful selection of the 293T-cathB1 cell line.

To further identify the ectopic expression of Ac-cathB-1 in the 293T-cathB1 cells, total RNA and protein were extracted from the two cell lines, 293T-cathB1 and 293T-GFP. With the specific primers P4, a fragment of *Ac-cathB-1* was amplified only in samples of 293T-cathB1 cells (Fig. 1D, top panel). Furthermore, single bands that were recognized by western blot assay with the homemade antiserum mentioned above demonstrated that the target protein was expressed highly in the 293T-cathB1 cell line (Fig. 1D, lower panel).

**Figure 2.**
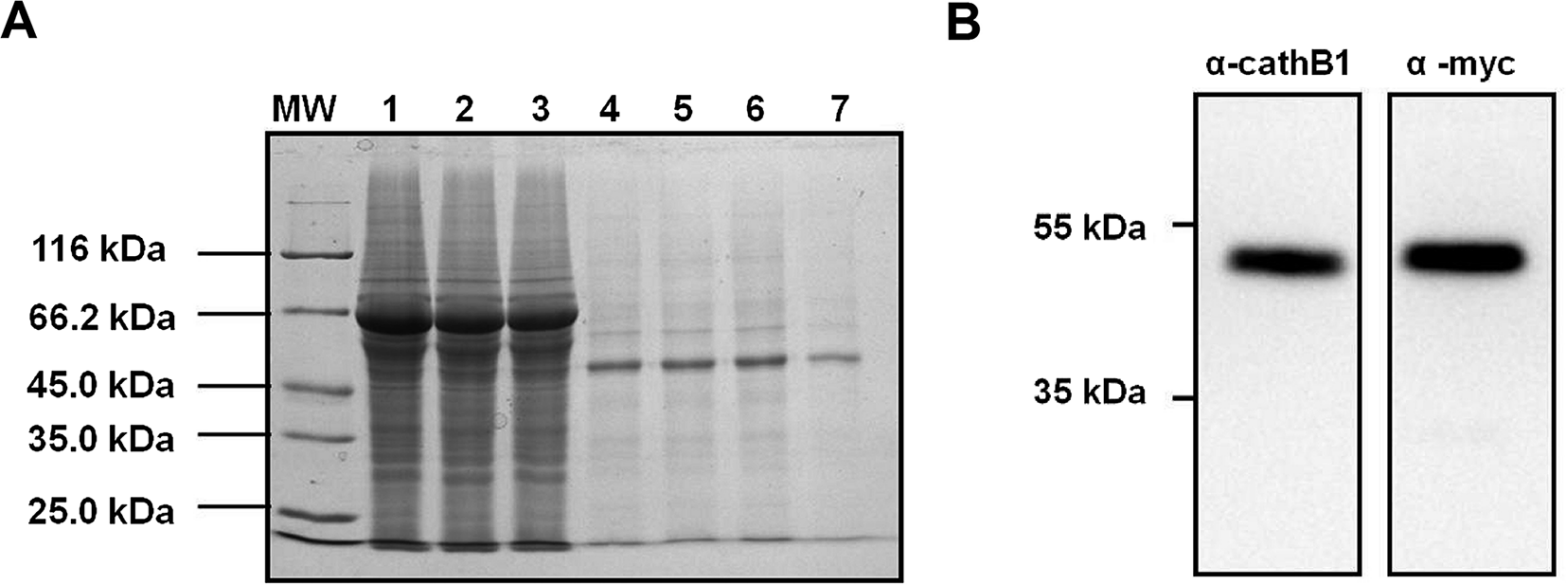
Purification and identification of rAc-cathB-1. (A) Purification of rAc-cathB-1 from the cell culture supernatant. Lanes: MW, molecular weight markers (listed in kDa on the side); 1, concentrated culture supernatant in binding buffer; 2, column flowthrough; 3, binding buffer wash; 4–6, successive eluate fractions (during the elution step, the eluate was collected in one 5 ml tube after another); 7, eluate from gel filtration. (B) Identification of purified rAc-cathB-1 by western blot with an antiserum against Ac-cathB-1 expressed in *E. coli* and an anti-Myc antibody.

The 293T-cathB1 cell line was cultured and rAc-cathB-1 was purified from the culture medium at a yield of 1.4 mg/L culture medium by Ni-affinity chromatography and gel filtration (Fig. 2A). In the fractions from the nickel resin, the purified protein migrated with an apparent molecular mass of 46 kDa (lane 4–6), and fraction through gel filtration displayed a single band at the same position (around 46 kDa) on the gel (lane 7), suggesting further purification (Fig. 2A). Purified rAc-cathB-1 was recognized at the expected position by both the homemade antiserum against prokaryotic rAc-cathB-1and anti-Myc antibody (Fig. 2B). To further ensure that the end product was rAc-cathB-1, the amino acid sequence was determined by MS. The mass spectrum of one of the fragments of the purification product was found to be the peptide residues 142–161 (DQSSCGSCWAFGAVEAMSDR) of Ac-cathB-1, which was identical to the cysteine-containing active site of this protease determined by bioinformatics analysis [24]. This result confirmed that the purified protein was indeed rAc-cathB-1.

**Figure 3.**
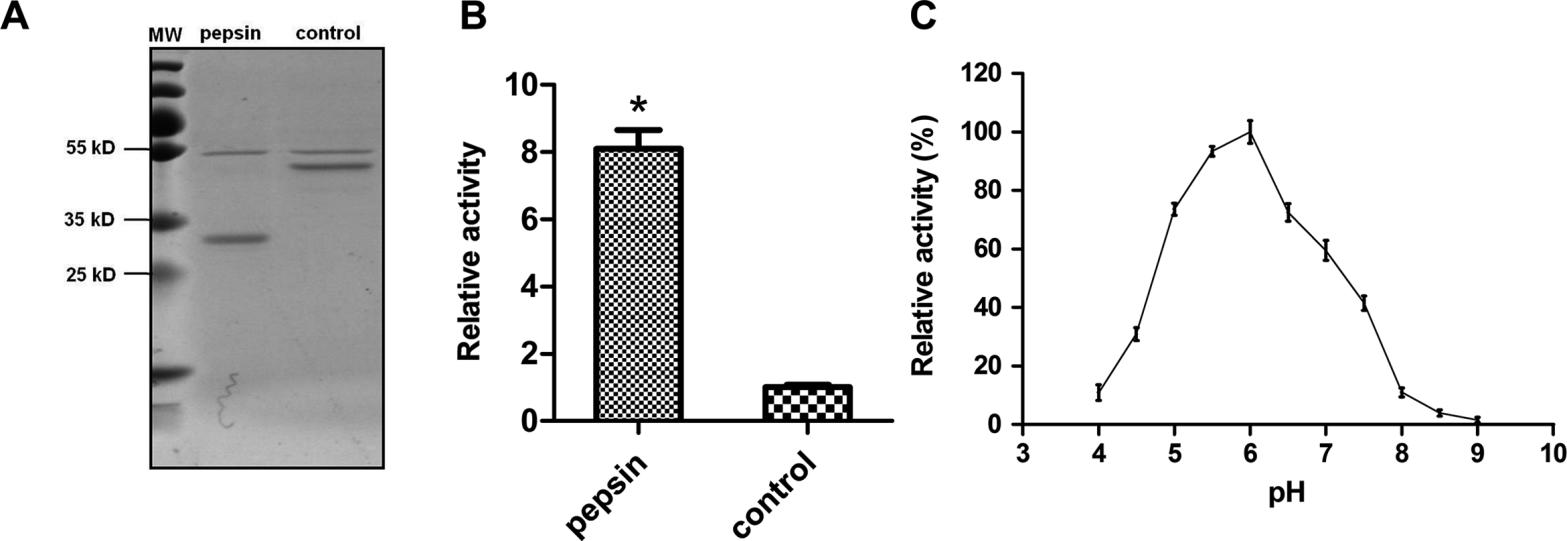
Activation and pH-dependence profile of rAc-cathB-1. (A) Processing of rAc-cathB-1. Purified rAc-cathB-1 (0.2 mg/mL) was incubated with activation solution or reference solution in a 2:1 (v/v) ratio. Mixtures were incubated at 37 °C for 30 min, and the reaction was stopped by addition of pepstatin A (Sigma-Aldrich) to a final concentration of 1 mM. Samples were analyzed on a 12% SDS-PAGE gel followed by Coomassie brilliant blue staining. MW, molecular weight markers; purified rAc-cathB-1 incubated with activation solution and reference solution as indicated. (B) Enzymatic activity assay. Z-Arg-Arg-7-amido-4-methylcoumarin hydrochloride was used for studying the activity of pepsin-treated rAc-cathB-1 and the control. The fluorescence was measured with excitation and emission wavelengths of 355 and 460 nm, respectively, and data were presented as relative activities, where activity of the control was taken as 1. (C) The pH-dependence profile of activated rAc-cathB-1. The assay was performed with the fluorescent substrate at a final concentration of 50 μM. Fluorescence was measured and data were presented as relative activities of activated rAc-cathB-1, where the highest activity at the pH optimum was taken as 100%. Asterisk (*), *P* < 0.05.

We then investigated the activation of rAc-cathB-1. In the presence of pepsin-HCl, rAc-cathB-1 was digested into a smaller peptide with a molecular mass of around 32 kDa (Fig. 3A). This result was similar to the cleavage of human cathepsin B [3, 31], indicating that pepsin may participate in the processing of Ac-cathB-1. Together with a 7.1-fold increase (*P* = 1.6 × 10^−3^, *P* < 0.05, by *t*-test) in hydrolytic activity on the fluorescent substrate in comparison with that of untreated rAc-cathB-1 (Fig. 3B), our results supported activation of the protease by pepsin. The pH-dependence profile of activated rAc-cathB-1 was studied. This protease exhibited a roughly bell-shaped pH profile from pH 4 to 8.5 with the pH optimum around pH 6.0 and showed much lower activity under a weak alkali environment (Fig. 3C).

**Figure 4.**
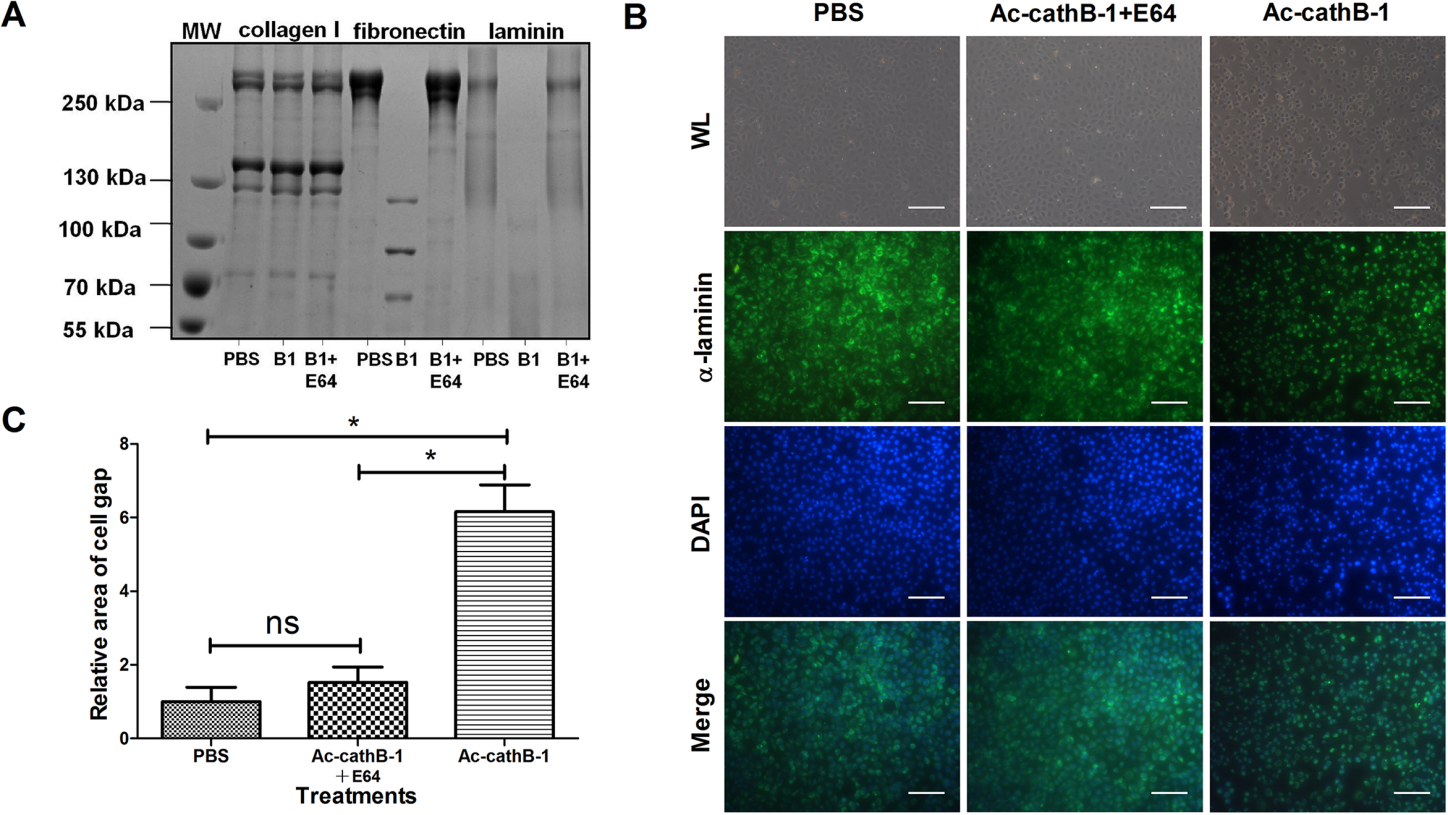
Assessment of the hydrolytic activity of activated rAc-cathB-1. (A) Activated rAc-cathB-1 completely degraded fibronectin and laminin but did not cleave type I collagen over a 12-h period. In the incubation buffer (pH 6.0), 10 μg of the respective substrates was treated with equal volumes of PBS, activated rAc-cathB-1, or activated rAc-cathB-1 plus E64 at 37 °C for 12 h. After incubation, all samples were analyzed on a 5% SDS-PAGE gel followed by Coomassie brilliant blue staining. Molecular weight markers (in kDa) are listed on the side. The substrate used for each experiment is indicated at the top of the gel, and the presence or absence of a recombinant protease or protease inhibitor is indicated at the bottom of each lane. (B) Influence of rAc-cathB-1 on IEC-6 monolayer. IEC-6 cells were grown to confluence and equal amounts of rAc-cathB-1 with or without E64 were applied to cells for 2 h. The blank was made of IEC-6 with PBS added. After incubation, the adherent IEC-6 partly rounded up and the integrity of the cell sheet was disrupted by activated rAc-cathB-1. The cytoplasm and ECM were then labeled with an anti-laminin antibody (green), and the nucleus was stained with DAPI. On the merged images, the dark regions represent the intercellular space. (C) Statistical analysis of the changes to the intercellular space of IEC-6 cells. The dark area was measured and analyzed. The difference between the means for each group of samples was estimated using one-way ANOVA followed by Duncan’s multiple comparison test. Asterisk (*), *P* < 0.05; WL, white light; *ns*: not significant; and bar = 100 μm.

To evaluate the bioactivity of activated rAc-cathB-1, it was incubated with various connective tissue proteins (Fig. 4). Unlike type I collagen (lane 3), fibronectin (lane 6) and laminin (lane 9) were readily digested, and this activity was inhibited by E64 (lane 7, 10). This indicates that activated rAc-cathB-1 was catalytically active against some of the extracellular matrix (ECM) components of connective tissue, with a substrate preference for fibronectin and laminin (Fig. 4A).

To investigate the influence of Ac-cathB-1 on small intestine epithelial cells, the activated recombinant protease was incubated with a confluent IEC-6 monolayer. Unlike the confluent and continuous cell sheet in the control, treatment with activated rAc-cathB-1 augmented intercellular space formation, and single cells partly rounded up from the adherent status (Fig. 4B, WL). Under the fluorescence microscope, the laminin-positive area and the nuclei displayed green and blue fluorescence, respectively, and the dark area in the merged image represents the intercellular space (Fig. 4B). To quantify the changes to the cell sheets, the dark area was measured, and the result showed that the three treatments differed significantly (*P* = 4.2 × 10^−5^, *P* < 0.05, by ANOVA). Activated rAc-cathB-1 exhibited an obvious effect on the dark area in comparison with the control (*P* = 1.6 × 10^−3^, *P* < 0.05), leading to a 5.15-fold increase (Fig. 4C). In the experiment where E64 was added, all the changes described above were partly blocked by the inhibition of enzymatic activity. These results indicate that activated rAc-cathB-1 possesses hydrolytic activity toward the ECM of IEC-6 and the activity could be specifically inhibited by E64.

**Figure 5.**
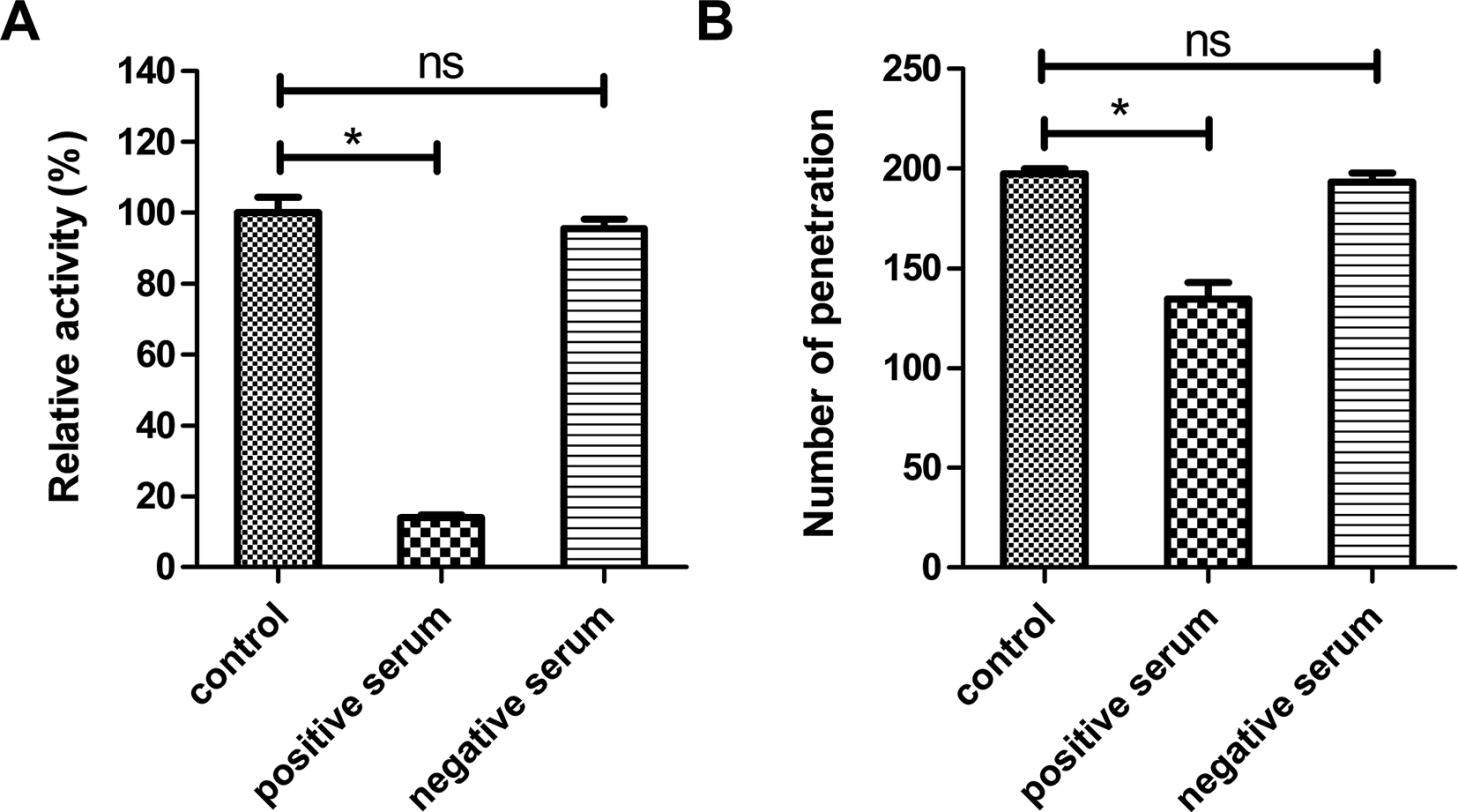
Effects of antiserum on the hydrolytic activity of activated rAc-cathB-1 and larval gut penetration. (A) The inhibition of activated rAc-cathB-1 by antiserum. One microgram of rAc-cathB-1 was activated and incubated with the positive serum (6.0 μg) or negative serum (6.0 μg) for 30 min, respectively, prior to assessment of the degradation of Z-RR-AMC. The hydrolytic reaction was performed at 37 °C for 30 min and the fluorescence of released AMC was measured. All data were presented as relative activities of activated rAc-cathB-1, where the activity of the control (without serum treatment) was taken as 100%. (B) Inhibition of larval penetration ability. Two hundred L3 larvae were pretreated with the undiluted positive serum, the undiluted negative serum, or PBS at 37 °C for 30 min, respectively. The three groups of larvae were separately injected into lumens of rat gut sacks and kept in sterilized Tyrode’s solution at 37 °C for 3 h. Each trial was conducted in triplicate and the numbers of larvae remaining in the gut lumen were counted. Numbers of L3 that penetrated the isolated gut were calculated and presented as indicated. Asterisk (*), *P* < 0.05; *ns*, not significant.

To study the relationship between Ac-cathB-1 and larval gut penetration, the effect of antiserum on proteolytic activity of activated rAc-cathB-1 was assessed in vitro (Fig. 5A). In comparison with the control, pretreatment of activated rAc-cathB-1 with the positive serum inhibited the hydrolysis of the fluorescent substrate by 86.1% (*P* = 4.7 × 10^−6^, *P* < 0.05, by *t*-test). An insignificant reduction was detected in the group pretreated with normal mouse serum (*P* = 0.23, *P* > 0.05, by *t*-test). Further, L3 were preincubated with positive serum, negative serum, and PBS before the isolated gut invasion assay (Fig. 5B). Compared with the control (PBS treated), positive serum incubation prevented 31.8% (*P* = 3.0 × 10^−3^, *P* < 0.05, by *t*-test) of L3 from penetrating the intestinal wall in vitro, while negative mouse serum resulted in an insignificant reduction in larval migration (*P* = 0.25, *P* > 0.05, by *t*-test). The partial inhibition suggests that other proteases may be involved in this process.

## Discussion

Although RNAi was achieved in diverse parasitic organisms [18, 39], and transgenic and knockout technology in some kinetoplastid parasites [10, 23, 26], many parasitic nematodes were proven refractory to genetic manipulation [21]. As for *A. cantonensis*, no successful application of genetic modification has been reported up to now. Consequently, heterologous expression of genes in this species was helpful for function explorations of specific gene products. The prokaryotic expression system was thought to have advantages such as higher expression levels, ease of operation, and low cost [28]. However, many gene products were often expressed in inclusion bodies in *E. coli*, without desired activity. To date, various eukaryotic expression systems have been explored for bioactive products, and large-scale production could be employed in yeast [16] and insect [25, 36] cells. However, proteins expressed in yeast were often defective in proper glycosylation and the baculovirus system results in insect cell death and lysis. Hence, we attempted to express these proteins in a secretable form in a lentiviral system for desired solubility and biological activity, considering, also, the low expression levels of cytoplasmic proteins in mammalian cells. We thus generated a modified LV and the gene of interest could be inserted into the expression cassette to produce a secreted fusion protein with a His tag for downstream purification. With the infection of the recombinant virus and subsequent selection, the modified expression cassette could integrate into the host genome and positive cells could express the target gene continuously.

Many parasitic proteases have hydrolytic activity to facilitate host invasion by degrading components of the ECM. In our previous study, native Ac-cathB-1 was found to be present in the digestive tract as well as excretory pore and excretory tube and predicted to be a component of the excretory/secretory products involved in tissue invasion. However, this protease was formerly expressed in an inactive form without correct folding and post-translational modification. In this work, we attempted to obtain rAc-cathB-1 by creating a stable 293T-cathB1 cell line using the modified lentiviral expression system. Although the charged His residues of some His-tagged recombinant proteins fail to bind the nickel resin due to spatial inaccessibility of the charged His to the immobilized nickel [36], rAc-cathB-1 was recognized in the eluate fractions by Western blot and further MS analysis, indicating successful recombinant expression and purification. In previous studies, the cathepsin Bs were found to be polypeptides consisting of a signal peptide, a propeptide, and a catalytic domain [3], and an aspartic protease was reported to be involved in the proteolytic trimming of these proenzymes [30]. Consequently, we investigated the involvement of pepsin in the processing of rAc-cathB-1. Together with the cleavage of the 46-kDa pro-cathepsin B polypeptide and increase of proteolytic activity, we confirmed that pepsin was capable of activating the proenzyme form of Ac-cathB-1. With the activated rAc-cathB-1, the pH-dependence profile was determined. Unlike the low levels of hydrolysis of other *A. cantonensis* cathepsin B family member at neutral pH [4, 8, 22], activated rAc-cathB-1 had relatively high activity at pH 6.0–7.0. Activity in this pH range makes it possible for activated rAc-cathB-1 to function under the neutral pH conditions of the small intestine.

In the case of *A. cantonensis*, intestinal penetration of the infective larvae is critical for its infection, and parasitic proteases possibly play key roles in this process. We thus investigated the digestive ability of activated rAc-cathB-1 by incubation with connective tissue proteins. We found the enzyme was catalytically active against fibronectin and laminin, but not against type I collagen. The substrate preference suggests the possible involvement of other proteases in the degradation of type I collagen and other ECM components. As an alternative approach to study the hydrolytic activity of activated rAc-cathB-1 toward the host intestine, we observed the effect of activated rAc-cathB-1 on an IEC-6 epithelial cell monolayer. Consistent with the digestive activity against matrix proteins, activated rAc-cathB-1 was able to alter the shape of the IEC-6 cells as well as disrupt the integrity of the cell sheet, suggesting that it possesses hydrolytic activity against the ECM of IEC-6, resulting in broadening of the intercellular space, possibly aiding gut penetration. However, no similar effect was found in 293T-cathB1 cells, partly because rAc-cathB-1 was expressed as a proenzyme form with low activity. The addition of serum in rAc-cathB-1-producing cells may also inhibit the hydrolytic activity of rAc-cathB-1. To confirm the role of this protease in gut penetration, we studied the alteration in larval penetration ability by inhibiting Ac-cathB-1 with the positive serum. Even though this polyclonal antiserum was produced against a non-active rAc-cathB-1, it was able to inhibit the hydrolytic ability of the activated rAc-cathB-1 to Z-RR-AMC *in vitro*. Although the neutral pH of the small intestine lumen was different from the optimum pH of activated rAc-cathB-1, our results supported that this protease displayed relatively high activity in pH 7.0. The ability of this antiserum to inhibit larval gut penetration *in vitro* suggested the association between Ac-cathB-1 and gut penetration.

In summary, we constructed a modified LV-driven expression of the target gene in a secretable form. Activated rAc-cathB-1 was shown to be active against ECM proteins and affect the shape and integrity of an epithelial cell line, indicating the probable function of this enzyme in host intestinal invasion. Moreover, inhibition of native Ac-cathB-1 with specific antiserum resulted in attenuation of larval penetration ability, indicating a role for Ac-cathB-1 in rat gut invasion by L3. Hence, the LV-based mammalian expression system presented here is a good alternative for stable expression of parasite proteins.

## References

[1] Alicata JE. 1991 The discovery of *Angiostrongylus cantonensis* as a cause of human eosinophilic meningitis. Parasitology Today, 7(6), 151–153. 15463478 10.1016/0169-4758(91)90285-v

[2] Antoniou MN, Skipper KA, Anakok O. 2013 Optimizing retroviral gene expression for effective therapies. Human Gene Therapy, 24(4), 363–374. 23517535 10.1089/hum.2013.062

[3] Brömme D, Wilson S. 2011 Role of cysteine cathepsins in extracellular proteolysis, in Extracellular Matrix Degradation, Parks WC, Mecham RP, Editors Springer: Berlin Heidelberg p. 23–51.

[4] Cheng M, Yang X, Li Z, He H, Qu Z, He A, Wu Z, Zhan X. 2012 Cloning and characterization of a novel cathepsin B-like cysteine proteinase from *Angiostrongylus cantonensis*. Parasitology Research, 110(6), 2413–2422. 22215189 10.1007/s00436-011-2780-y

[5] Costa SJ, Almeida A, Castro A, Domingues L, Besir H. 2013 The novel Fh8 and H fusion partners for soluble protein expression in *Escherichia coli*: a comparison with the traditional gene fusion technology. Applied Microbiology and Biotechnology, 97(15), 6779–6791. 23160981 10.1007/s00253-012-4559-1

[6] Diao Z, Yin C, Qi H, Wang J. 2011 International symposium on *Angiostrongylus* and Angiostrongyliasis, 2010. Emerging Infectious Diseases, 17(7), e1. 21762566 10.3201/eid1707.102038 PMC3381375

[7] Dubreuil G, Deleury E, Magliano M, Jaouannet M, Abad P, Rosso MN. 2011 Peroxiredoxins from the plant parasitic root-knot nematode, *Meloidogyne incognita*, are required for successful development within the host. International Journal for Parasitology, 41(3–4), 385–396. 21145323 10.1016/j.ijpara.2010.10.008

[8] Han YP, Li ZY, Li BC, Sun X, Zhu CC, Ling XT, Zheng HQ, Wu ZD, Lv ZY. 2011 Molecular cloning and characterization of a cathepsin B from *Angiostrongylus cantonensis*. Parasitology Research, 109(2), 369–378. 21344211 10.1007/s00436-011-2264-0

[9] Jenkins MC, O’Brien CN, Fuller L, Mathis GF, Fetterer R. 2014 A rapid method for determining salinomycin and monensin sensitivity in *Eimeria tenella*. Veterinary Parasitology, 206(3–4), 153–158. 25312355 10.1016/j.vetpar.2014.09.017

[10] Kangethe RT, Boulange AF, Coustou V, Baltz T, Coetzer TH. 2012 *Trypanosoma brucei brucei* oligopeptidase B null mutants display increased prolyl oligopeptidase-like activity. Molecular and Biochemal Parasitology, 182(1–2), 7–16. 10.1016/j.molbiopara.2011.11.007 22123425

[11] Lai CH, Yen CM, Chin C, Chung HC, Kuo HC, Lin HH. 2007 Eosinophilic meningitis caused by *Angiostrongylus cantonensis* after ingestion of raw frogs. American Journal of Tropical Medicine and Hygiene, 76(2), 399–402. 17297055

[12] Lipps G, Fullkrug R, Beck E. 1996 Cathepsin B of *Schistosoma mansoni*. Purification and activation of the recombinant proenzyme secreted by *Saccharomyces cerevisiae*. Journal of Biological Chemistry, 271(3), 1717–1725. 8576174 10.1074/jbc.271.3.1717

[13] List K, Hoyer-Hansen G, Ronne E, Dano K, Behrendt N. 1999 Different mechanisms are involved in the antibody mediated inhibition of ligand binding to the urokinase receptor: a study based on biosensor technology. Journal of Immunological Methods, 222(1–2), 125–133. 10022379 10.1016/s0022-1759(98)00189-6

[14] Liu K, Wang H, Long Y, Ye J, Yuan L. 2012 Coordinate lentiviral expression of Cre recombinase and RFP/EGFP mediated by FMDV 2A and analysis of Cre activity. Journal of Cellular Biochemistry, 113(9), 2909–2919. 22532014 10.1002/jcb.24168

[15] Lv S, Zhang Y, Liu HX, Hu L, Yang K, Steinmann P, Chen Z, Wang LY, Utzinger J, Zhou XN. 2009 Invasive snails and an emerging infectious disease: results from the first national survey on *Angiostrongylus cantonensis* in China. PLoS Neglected Tropical Diseases, 3(2), e368. 19190771 10.1371/journal.pntd.0000368 PMC2631131

[16] Macauley-Patrick S, Fazenda ML, McNeil B, Harvey LM. 2005 Heterologous protein production using the *Pichia pastoris* expression system. Yeast, 22(4), 249–270. 15704221 10.1002/yea.1208

[17] Martinez-Salas E. 1999 Internal ribosome entry site biology and its use in expression vectors. Current Opinion in Biotechnology, 10(5), 458–464. 10508627 10.1016/s0958-1669(99)00010-5

[18] Maule AG, McVeigh P, Dalzell JJ, Atkinson L, Mousley A, Marks NJ. 2011 An eye on RNAi in nematode parasites. Trends in Parasitology, 27(11), 505–513. 21885343 10.1016/j.pt.2011.07.004

[19] McCabe RE, Yu GS, Conteas C, Morrill PR, McMorrow B. 1991 In vitro model of attachment of *Giardia intestinalis* trophozoites to IEC-6 cells, an intestinal cell line. Antimicrobial Agents and Chemotherapy, 35(1), 29–35. 1901700 10.1128/aac.35.1.29 PMC244937

[20] McGonigle L, Mousley A, Marks NJ, Brennan GP, Dalton JP, Spithill TW, Day TA, Maule AG. 2008 The silencing of cysteine proteases in *Fasciola hepatica* newly excysted juveniles using RNA interference reduces gut penetration. International Journal for Parasitology, 38(2), 149–155. 18048044 10.1016/j.ijpara.2007.10.007

[21] Morales ME, Mann VH, Kines KJ, Gobert GN, Fraser MJ Jr, Kalinna BH, Correnti JM, Pearce EJ, Brindley PJ. 2007 piggyBac transposon mediated transgenesis of the human blood fluke, *Schistosoma mansoni*. FASEB Journal, 21(13), 3479–3489. 17586730 10.1096/fj.07-8726com

[22] Morassutti AL, Graeff-Teixeira C. 2012 Interface molecules of *Angiostrongylus cantonensis*: their role in parasite survival and modulation of host defenses. International Journal of Inflammation, 2012, 512097. 22536544 10.1155/2012/512097 PMC3321291

[23] Morrison LS, Goundry A, Faria MS, Tetley L, Eschenlauer SC, Westrop GD, Dostalova A, Volf P, Coombs GH, Lima AP, Mottram JC. 2012 Ecotin-like serine peptidase inhibitor ISP1 of *Leishmania major* plays a role in flagellar pocket dynamics and promastigote differentiation. Cellular Microbiology, 14(8), 1271–1286. 22486816 10.1111/j.1462-5822.2012.01798.x PMC3440592

[24] Ni F, Wang Y, Zhang J, Yu L, Fang W, Luo D. 2012 Cathepsin B-like and hemoglobin-type cysteine proteases: stage-specific gene expression in *Angiostrongylus cantonensis*. Experimental Parasitology, 131(4), 433–441. 22668746 10.1016/j.exppara.2012.05.014

[25] Otsuki T, Dong J, Kato T, Park EY. 2013 Expression, purification and antigenicity of *Neospora caninum*-antigens using silkworm larvae targeting for subunit vaccines. Veterinary Parasitology, 192(1–3), 284–287. 23102762 10.1016/j.vetpar.2012.09.038

[26] Peng D, Kurup SP, Yao PY, Minning TA, Tarleton RL. 2015 CRISPR-Cas9-mediated single-gene and gene family disruption in *Trypanosoma cruzi*. MBio, 6(1), e02097–14. 25550322 10.1128/mBio.02097-14 PMC4281920

[27] Pillay D, Boulange AF, Coustou V, Baltz T, Coetzer TH. 2013 Recombinant expression and biochemical characterisation of two alanyl aminopeptidases of *Trypanosoma congolense*. Experimental Parasitology, 135(4), 675–684. 24177338 10.1016/j.exppara.2013.10.005

[28] Rosano GL, Ceccarelli EA. 2014 Recombinant protein expression in *Escherichia coli*: advances and challenges. Frontiers in Microbiology, 5, 172. 24860555 10.3389/fmicb.2014.00172 PMC4029002

[29] Selkirk ME, Huang SC, Knox DP, Britton C. 2012 The development of RNA interference (RNAi) in gastrointestinal nematodes. Parasitology, 139(5), 605–612. 22459433 10.1017/S0031182011002332

[30] Shirahama-Noda K, Yamamoto A, Sugihara K, Hashimoto N, Asano M, Nishimura M, Hara-Nishimura I. 2003 Biosynthetic processing of cathepsins and lysosomal degradation are abolished in asparaginyl endopeptidase-deficient mice. Journal of Biological Chemistry, 278(35), 33194–33199. 12775715 10.1074/jbc.M302742200

[31] Smooker PM, Jayaraj R, Pike RN, Spithill TW. 2010 Cathepsin B proteases of flukes: the key to facilitating parasite control? Trends in Parasitology, 26(10), 506–514. 20580610 10.1016/j.pt.2010.06.001

[32] Stecker K, Koschel A, Wiedenmann B, Anders M. 2009 Loss of Coxsackie and adenovirus receptor downregulates alpha-catenin expression. British Journal of Cancer, 101(9), 1574–1579. 19773761 10.1038/sj.bjc.6605331 PMC2778516

[33] Viney ME, Thompson FJ. 2008 Two hypotheses to explain why RNA interference does not work in animal parasitic nematodes. International Journal for Parasitology, 38(1), 43–47. 18028931 10.1016/j.ijpara.2007.08.006

[34] Wang QP, Lai DH, Zhu XQ, Chen XG, Lun ZR. 2008 Human angiostrongyliasis. Lancet Infectious Diseases, 8(10), 621–630. 10.1016/S1473-3099(08)70229-9 18922484

[35] Weight CM, Carding SR. 2012 The protozoan pathogen *Toxoplasma gondii* targets the paracellular pathway to invade the intestinal epithelium. Annals of the New York Academy of Sciences, 1258, 135–142. 22731726 10.1111/j.1749-6632.2012.06534.x

[36] Williamson AL, Lustigman S, Oksov Y, Deumic V, Plieskatt J, Mendez S, Zhan B, Bottazzi ME, Hotez PJ, Loukas A. 2006 *Ancylostoma caninum* MTP-1, an astacin-like metalloprotease secreted by infective hookworm larvae, is involved in tissue migration. Infection and Immunity, 74(2), 961–967. 16428741 10.1128/IAI.74.2.961-967.2006 PMC1360348

[37] Xiao H, Bryksa BC, Bhaumik P, Gustchina A, Kiso Y, Yao SQ, Wlodawer A, Yada RY. 2014 The zymogen of plasmepsin V from *Plasmodium falciparum* is enzymatically active. Molecular and Biochemical Parasitology, 197(1–2), 56–63. 25447707 10.1016/j.molbiopara.2014.10.004 PMC6310130

[38] Yu C, Wang Y, Zhang J, Fang W, Luo D. 2014 Immunolocalization and developmental expression patterns of two cathepsin B proteases (AC-cathB-1, -2) of *Angiostrongylus cantonensis*. Experimental Parasitology, 144C, 27–33. 24929149 10.1016/j.exppara.2014.06.008

[39] Zawadzki JL, Kotze AC, Fritz JA, Johnson NM, Hemsworth JE, Hines BM, Behm CA. 2012 Silencing of essential genes by RNA interference in *Haemonchus contortus*. Parasitology, 139(5), 613–629. 22348596 10.1017/S0031182012000121

